# SARS-CoV-2 Viral Entry Proteins in Hyperandrogenemic Female Mice: Implications for Women with PCOS and COVID-19

**DOI:** 10.3390/ijms22094472

**Published:** 2021-04-25

**Authors:** Alexandra M. Huffman, Samar Rezq, Jelina Basnet, Licy L. Yanes Cardozo, Damian G. Romero

**Affiliations:** 1 Department of Cell and Molecular Biology, University of Mississippi Medical Center, Jackson, MS 39216, USA; ahuffman@umc.edu (A.M.H.); srezq@umc.edu (S.R.); jbasnet@umc.edu (J.B.); lyanes@umc.edu (L.L.Y.C.); 2 Mississippi Center of Excellence in Perinatal Research, University of Mississippi Medical Center, Jackson, MS 39216, USA; 3 Women’s Health Research Center, University of Mississippi Medical Center, Jackson, MS 39216, USA; 4 Cardio Renal Research Center, University of Mississippi Medical Center, Jackson, MS 39216, USA; 5 Department of Medicine, University of Mississippi Medical Center, Jackson, MS 39216, USA

**Keywords:** SARS-CoV-2, COVID-19, Polycystic Ovary Syndrome, androgens, angiotensin I converting enzyme 2, androgen receptor

## Abstract

SARS-CoV-2, the causative agent of COVID-19, infects host cells using the angiotensin I converting enzyme 2 (ACE2) as its receptor after priming by host proteases, including TMPRSS2. COVID-19 affects multiple organ systems, and male patients suffer increased severity and mortality. Polycystic Ovary Syndrome (PCOS) is the most common endocrine disorder in reproductive-age women and is characterized by hyperandrogenism, ovulatory dysfunction, and polycystic ovarian morphology. PCOS is associated with obesity and cardiometabolic comorbidities, both being risk factors associated with severe COVID-19 pathology. We hypothesize that elevated androgens in PCOS regulate SARS-CoV-2 entry proteins in multiple tissues increasing the risk for this population. Female mice were treated with dihydrotestosterone (DHT) for 90 days. Body composition was measured by EchoMRI. Fasting glucose was determined by an enzymatic method. mRNA and protein levels of ACE2, Tmprss2, Cathepsin L, Furin, Tmprss4, and Adam17 were quantified by RT-qPCR, Western-blot, or ELISA in tissues, serum, and urine. DHT treatment increased body weight, fat and lean mass, and fasting glucose. Ace2 mRNA was upregulated in the lung, cecum, heart, and kidney, while downregulated in the brain by DHT. ACE2 protein was upregulated by DHT in the small intestine, heart, and kidney. The SARS-CoV-2 priming proteases Tmprss2, Cathepsin L, and Furin mRNA were upregulated by DHT in the kidney. ACE2 sheddase Adam17 mRNA was upregulated by DHT in the kidney, which corresponded with increased urinary ACE2 in DHT treated mice. Our results highlight the potential for increased cardiac, renal, and gastrointestinal dysfunction in PCOS women with COVID-19.

## 1. Introduction

The continuing COVID-2019 pandemic, caused by the Severe Acute Respiratory Syndrome Coronavirus 2 (SARS-CoV-2), was declared a public health emergency by the World Health Organization in January 2020. The SARS-CoV-2 virus has caused a global pandemic reaching over 215 countries and led to over 110 million cases. Until the most recent SARS-CoV-2 emergence, only two human coronaviruses had emerged in recent years. The 2002 SARS-CoV, which shares a 76% amino acid sequence homology to SARS-CoV-2, had a 10% case fatality rate and originated in China. Secondly, 2012 Middle East Respiratory Syndrome Coronavirus (MERS-CoV) had a case-fatality rate of 35% and originated from the Middle East [1]. Most known human betacoronaviruses primarily infect the nasopharynx, but other symptoms frequently observed are headache, sore throat, fatigue, and gastrointestinal disruption. All three viruses have zoonotic origins. In addition to age, identifiable risk factors in susceptibility for the severity of the two previous outbreaks are similar to SARS-CoV-2 and include type 2 diabetes, renal disease, and hypertension [2,3].

Angiotensin-converting enzyme 2 (ACE2), a transmembrane, endothelium-bound type I glycoprotein, binds to SARS-CoV-2 Spike (S) protein to guide viral entry into the host cell, acting as the SARS-CoV-2 receptor [4]. It is hypothesized that the increased affinity of the SARS-CoV-2 receptor binding domain compared with that of SARS-CoV by the human ACE2 receptor allows for greater human transmission owing to increased binding affinity [5]. Viral S-protein must be cleaved by host proteases to become active [1]. This priming happens over multiple cleavage events in several different sites of the viral glycoprotein by the host’s own proteases. The transmembrane serine protease 2 (TMPRSS2) is the main protease that cleaves SARS-CoV-2 S-protein to induce viral membrane fusion and cell entry. However, the SARS-CoV-2 S-protein contains a polybasic cleavage site that is capable of undergoing processing by other proteases such as furin and cathepsin L [4]. Interestingly, ACE2 shows sexual dimorphic expression; male mice have increased renal and lung Ace2 protein expression compared with females [6,7]. Similarly, primary human airway cells from males have increased ACE2 protein expression compared with cells from females [8]. Additionally, TMPRSS2 protein expression is upregulated by androgens in androgen-sensitive human prostate adenocarcinoma cells [9]. Moreover, Ace2 and Tmprss2 mRNA are increased by testosterone, an androgen that can be converted to estrogens, in the bronchial cells of female mice [10]. Those previous reports suggest that SARS-CoV-2 entry proteins could be modulated by androgens in multiple tissues.

Androgen action and sensitivity in SARS-CoV-2 target tissues have been hypothesized as a determinant of COVID-19 disease severity [11]. Men have disproportionally higher case fatality and a higher rate of severity of COVID-19 requiring hospitalization [12,13]. Moreover, male mice have an enhanced susceptibility to the SARS-CoV [14]. It has been suggested that androgens may be a contributing factor to sex differences observed in COVID-19 outcomes. Males with androgenic alopecia, or male pattern hair loss, a sign of elevated androgens were more likely to be hospitalized for COVID-19 than normal males, suggesting that increased androgens may be associated with a more severe outcome [15,16]. These and other studies suggest that androgens may help explain the increased severity and mortality observed in males in COVID-19.

Polycystic ovary syndrome (PCOS) is the most common endocrine disorder in reproductive-age women affecting 5–20% of women worldwide [17,18]. PCOS is usually diagnosed by the Rotterdam criteria, which requires the presence of at least two of the following three criteria: hyperandrogenism (biochemical or clinical), ovulatory dysfunction (oligo- or anovulation), and polycystic ovary morphology [19]. Biochemical elevation in circulating androgens, or hyperandrogenemia, is present in ~80% of all women with PCOS [20,21]. A recent study showed that in a cohort of patients with COVID-19 admitted to the Intensive Care Unit, the majority (60%) of female patients had elevated testosterone [22]. Moreover, a recent cross-sectional case-control study showed that COVID-19 positive outpatient women with PCOS had a higher incidence of COVID-19-associated symptoms such as low-grade fever, anosmia, ageusia, dry cough, among others, when compared with non-hyperandrogenemic COVID-19 positive women [23]. A large epidemiological study of more than 20,000 women with PCOS, matched 1:4 with control women, found a 36% increase risk for COVID-19 compared to controls after adjusting for BMI, age, and impaired glucose regulation [24]. This study further highlights the relevance to this population and the need for heightened focus. However, those relationships have not been evaluated on a larger scale to assess hospitalizations or death. Interestingly, circulating androgens in women with PCOS are positively associated with the severity of metabolic dysfunction and obesity [25,26,27,28], both being well-recognized comorbidities in the severity of COVID-19 [29]. 

The purpose of this study is to analyze the role of hyperandrogenism in the expression regulation of the SARS-CoV-2 viral entry proteins in a well-established mouse model of PCOS [30]. COVID-19 symptoms include acute respiratory distress, confusion and headache, muscle pain, gastrointestinal disruption, chest pain and pressure, and renal dysfunction, among others. We analyzed SARS-CoV-2 viral entry proteins in multiple organs involved in COVID-19 pathology. Our study may help elucidate tissue-specific vulnerabilities of women with PCOS to SARS-CoV-2 infection and COVID-19-associated pathologies.

## 2. Results

### 2.1. Dihydrotestosterone Treatment Caused Obesity and Glucose Homeostasis Dysregulation in Female Mice

**Figure 1:**
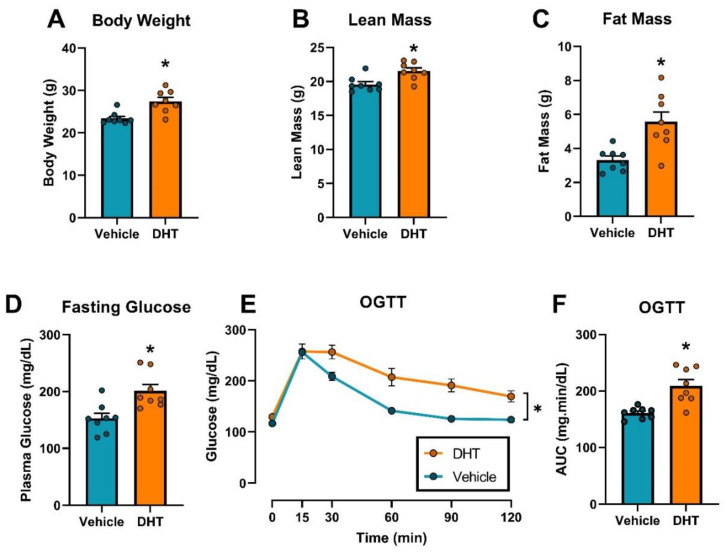
Effect of DHT on body weight and composition, and glucose homeostasis. (**A**) Body weight (*n* = 8/group). (**B**) Fat mass by EchoMRI (*n* = 8/group). (**C**) Lean mass by EchoMRI (*n* = 8/group). (**D**) Fasting glucose (*n* = 8/group). (**E**) Time-course of Oral glucose tolerance test (OGTT) (*n* = 8/group). (**F**) Area under the curve (AUC) from OGTT (*n* = 8/group). Data analyzed by *t*-test. *: *p* < 0.05.

We first validated the hyperandrogenemic female mice as an experimental model of PCOS that resembles the human PCOS phenotype. Hyperandrogenemic females had increased body weight (27.43 ± 0.92 vs. 23.42 ± 0.50 g, Figure 1A), lean mass (21.56 ± 0.45 vs. 19.58 ± 0.39 g, Figure 1B) and increased fat mass (5.57 ± 0.58 vs. 3.32 ± 0.22 g, Figure 1C) compared to their vehicle counterparts. Dihydrotestosterone (DHT)-treated females showed altered glucose homeostasis, having increased fasting glucose (201.10 ± 11.11 vs. 152.80 ± 9.23 mg/dL, Figure 1D) and an increased area under the curve (209.2 ± 11.0 vs. 160.8 ± 3.5 mg.min/dL) by OGTT (Figure 1E,F). 

### 2.2. Androgen Receptor Is Expressed in Multiple Tissues in Female Mice

**Figure 2:**
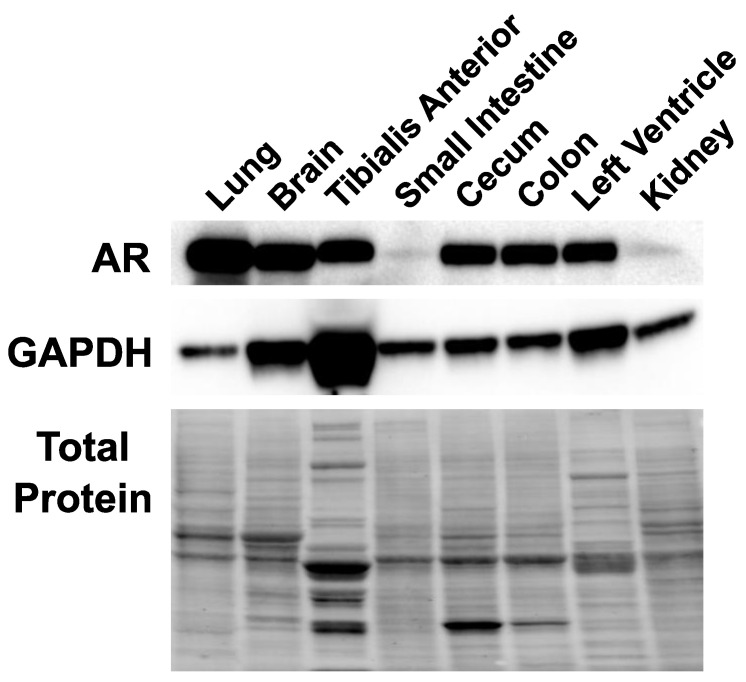
Androgen receptor protein expression in multiple tissues. Androgen receptor protein expression was analyzed by Western blot. Equal amounts of protein extracts (50 µg total protein) were analyzed for each tissue. GAPDH is shown to represent protein expression across tissues as a positive loading control. The total protein is shown by the use of Stain-Free total protein staining technology.

We analyzed androgen receptor protein expression in the tissues considered for SARS-CoV-2 entry proteins expression regulation to validate that those tissues could be subjected to androgen modulation. The androgen receptor protein was expressed in all tissues analyzed in untreated female mice (Figure 2). The androgen receptor protein was detected in the lung, brain, tibialis anterior, small intestine, cecum, colon, left ventricle, and kidney. These results suggest that SARS-CoV-2 entry proteins could be subjected to androgen regulation in all the aforementioned tissues. 

### 2.3. ACE2 Expression Is Modulated by DHT

**Figure 3:**
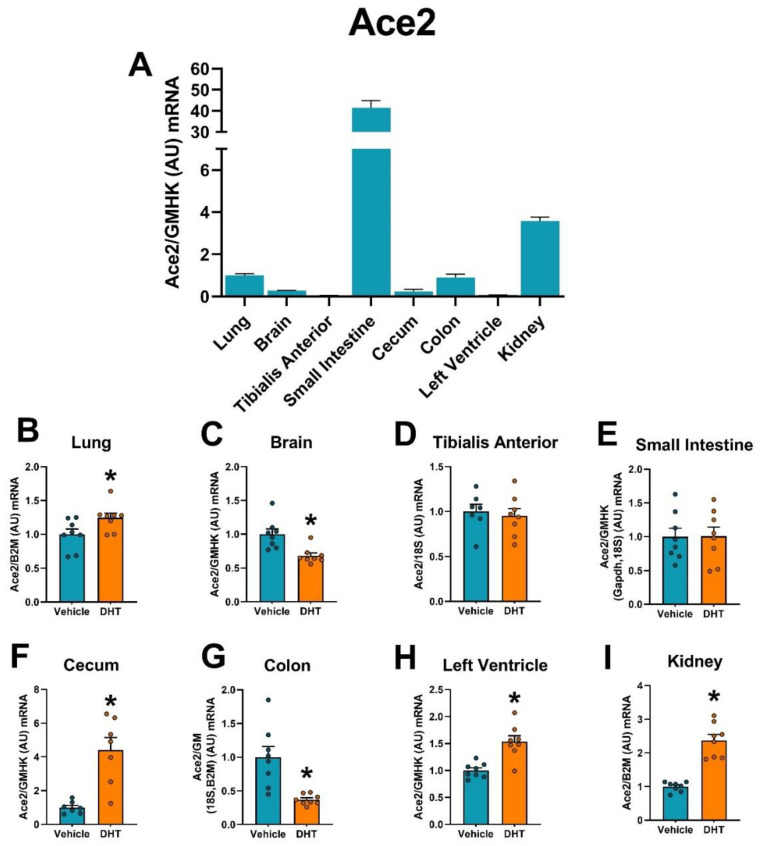
Ace2 mRNA expression regulation by DHT in different tissues. Ace2 mRNA expression was quantified by RT-qPCR. (**A**) Ace2 mRNA expression in control mice normalized to the geometric mean of all reference genes and expressed relative to lung tissue. (**B**–**I**) Ace2 mRNA expression in the lung (**B**), brain (**C**), tibialis anterior (**D**), small intestine (**E**), cecum (**F**), colon (**G**), left ventricle (**H**), and kidney (**I**) of control and DHT-treated mice normalized to the least variable reference gene or set of them and expressed relative to control mice. Data analyzed by *t*-test. *n* = 7–8/group. *: *p* < 0.05.

**Figure 4:**
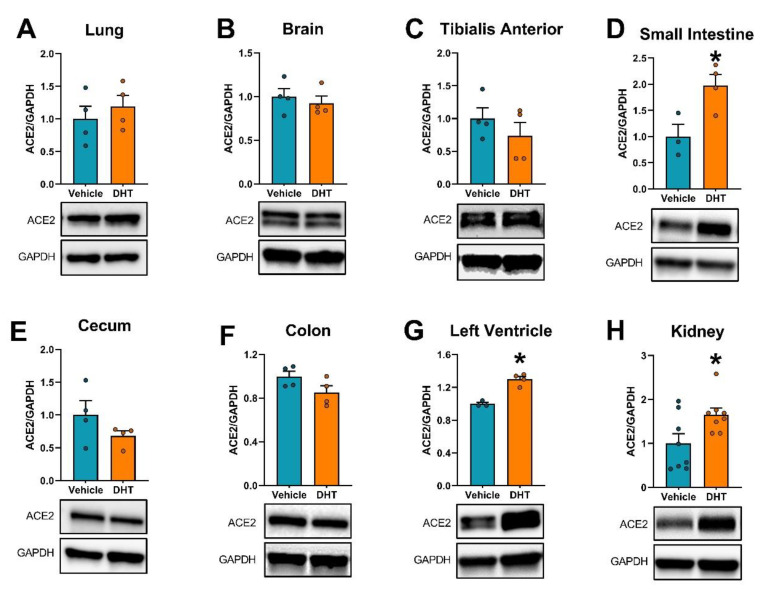
ACE2 protein expression regulation by DHT in different tissues. ACE2 protein expression was quantified by Western blot. ACE2 protein expression in the lung (**A**), brain (**B**), tibialis anterior (**C**), small intestine (**D**), cecum (**E**), colon (**F**), left ventricle (**G**), and kidney (**H**) of control and DHT-treated mice. Protein expression was normalized to GAPDH and expressed relative to control mice. Data analyzed by *t*-test. *n* = 3–8/group. *: *p* < 0.05.

We then analyzed the regulation of the SARS-CoV-2 receptor ACE2 mRNA and protein by androgens in multiple tissues. The small intestine was shown to have the greatest relative Ace2 mRNA expression compared with the other tissues, followed by the kidney (Figure 3A). Ace2 mRNA was upregulated in DHT-treated animals in the cecum (4.4-fold, Figure 3F), the left ventricle (1.54-fold, Figure 3H), and the kidney (2.37-fold, Figure 3I), and was downregulated in DHT-treated animals in the brain (0.68-fold, Figure 3C), and the colon (0.37-fold, Figure 3G). ACE2 protein expression was increased in the small intestine (1.98-fold, Figure 4D), the left ventricle (1.30-fold, Figure 4G), and the kidney (1.32-fold, Figure 4H).

### 2.4. Urinary ACE2 Protein Is Increased by DHT

**Figure 5:**
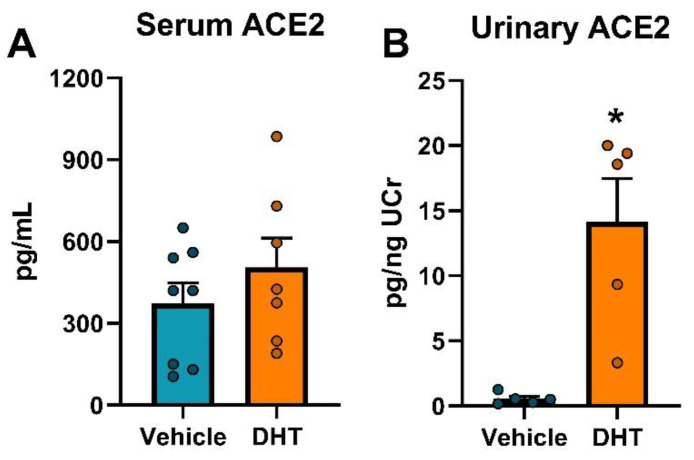
Serum and urinary ACE2 protein regulation by DHT. Serum (**A**, *n* = 7–8/group) and urinary ACE2 (**B**, *n* = 5/group) corrected by urinary creatinine. Data analyzed by *t*-test, with Welch’s correction for urinary ACE2. *: *p* < 0.05.

We, later on, analyzed the effect of excess androgens on soluble ACE2, both circulating and urinary. No significant effect of DHT treatment was observed in serum levels of ACE2 protein (Figure 5A). However, DHT-treated animals showed higher urinary ACE2 protein (14.13 ± 3.33 vs. 0.55 ± 0.19 pg/ng creatinine, Figure 5B). 

### 2.5. Proteases Expression Are Tissue-Specifically Modulated by DHT

**Figure 6:**
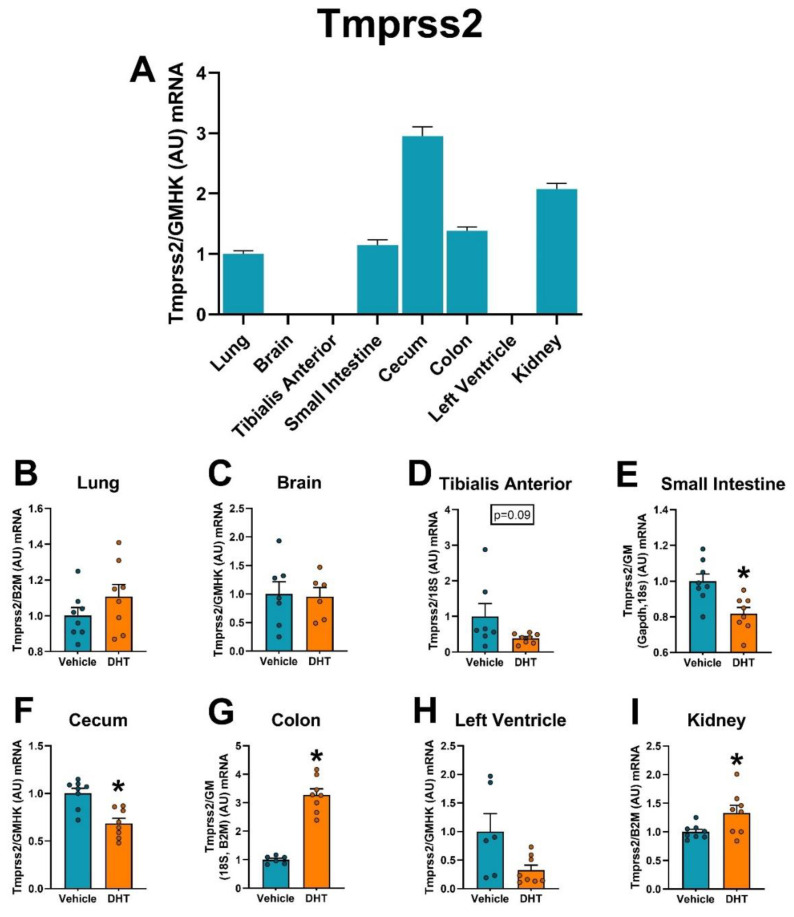
Tmprss2 mRNA expression regulation by DHT in different tissues. Tmprss2 mRNA expression was quantified by RT-qPCR. (**A**) Tmprss2 mRNA expression in control mice normalized to the geometric mean of all reference genes and expressed relative to lung tissue. (**B**–**I**) Tmprss2 mRNA expression in the lung (**B**), brain (**C**), tibialis anterior (**D**), small intestine (**E**), cecum (**F**), colon (**G**), left ventricle (**H**), and kidney (**I**) of control and DHT-treated mice normalized to the least variable reference gene or set of them and expressed relative to control mice. Data analyzed by *t*-test. *n* = 7–8/group. *: *p* < 0.05.

**Figure 7:**
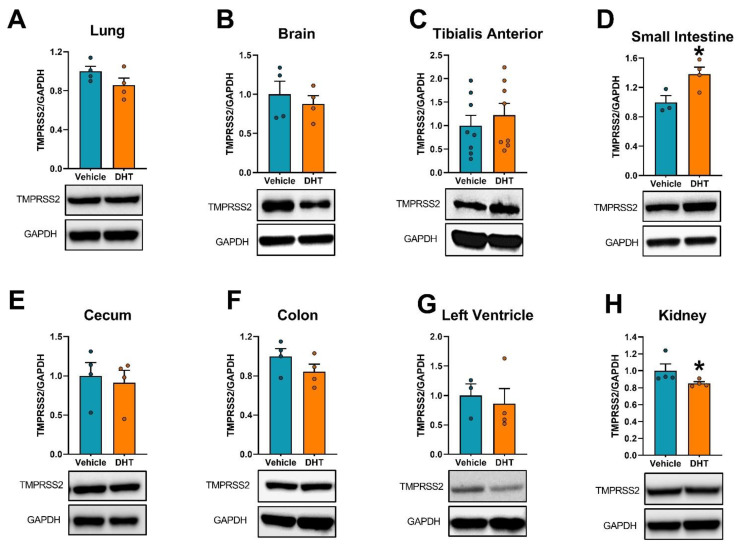
TMPRSS2 protein expression regulation by DHT in different tissues. TMPRSS2 protein expression was quantified by Western blot. TMPRSS2 protein expression in the lung (**A**), brain (**B**), tibialis anterior (**C**), small intestine (**D**), cecum (**E**), colon (**F**), left ventricle (**G**), and kidney (**H**) of control and DHT-treated mice. Protein expression was normalized to GAPDH and expressed relative to control mice. Data analyzed by *t*-test. *n* = 3–8/group. *: *p* < 0.05.

Finally, we analyzed the expression regulation of host cell’s proteases involved in SARS-CoV-2 cell entry (Tmprss2, cathepsin L, furin, and Tmprss4) and ACE2 regulation (Adam17) by androgens in multiple tissues. The highest expression of Tmprss2 mRNA was in the gastrointestinal tract and the kidney (Figure 6A). Tmprss2 mRNA was upregulated in DHT-treated animals in the colon (3.28-fold, Figure 6G) and the kidney (1.33-fold, Figure 6I). On the other hand, Tmprss2 mRNA was downregulated in DHT-treated animals in the small intestine (0.82-fold, Figure 6E) and the cecum (0.69-fold, Figure 6F). DHT treatment regulated TMPRSS2 protein levels by increases in the small intestine (1.38-fold, Figure 7D) and decreased levels in the kidney (0.92-fold, Figure 7H).

**Figure 8:**
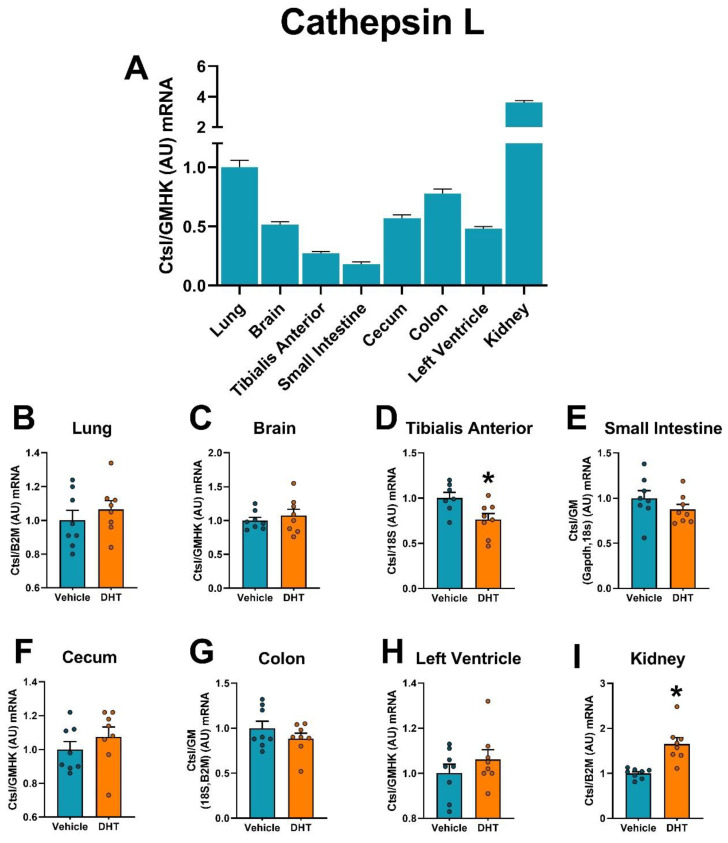
Cathepsin L mRNA expression regulation by DHT in different tissues. Cathepsin L mRNA expression was quantified by RT-qPCR. (**A**) Cathepsin L mRNA expression in control mice normalized to the geometric mean of all reference genes and expressed relative to lung tissue. (**B**–**I**) Cathepsin L mRNA expression in the lung (**B**), brain (**C**), tibialis anterior (**D**), small intestine (**E**), cecum (**F**), colon (**G**), left ventricle (**H**), and kidney (**I**) of control and DHT-treated mice normalized to the least variable reference gene or set of them and expressed relative to control mice. Data analyzed by *t*-test. *n* = 7–8/group. *: *p* < 0.05.

Cathepsin L mRNA had the highest expression in the kidney, compared with other tissues (Figure 8A). Cathepsin L mRNA expression was upregulated in DHT-treated animals in the kidney (1.65-fold, Figure 8I) and downregulated in the tibialis anterior muscle (0.76-fold, Figure 8D). Cathepsin L protein was measured in the left ventricle of the heart because it has been suggested as an alternative SARS-CoV-2 entry factor in that tissue to overcome the low levels of Tmprss2 expression [31]. DHT-treated animals showed a modest 0.85-fold tendency to decrease Cathepsin L protein levels in the left ventricle (Supplemental Figure S1).

**Figure 9:**
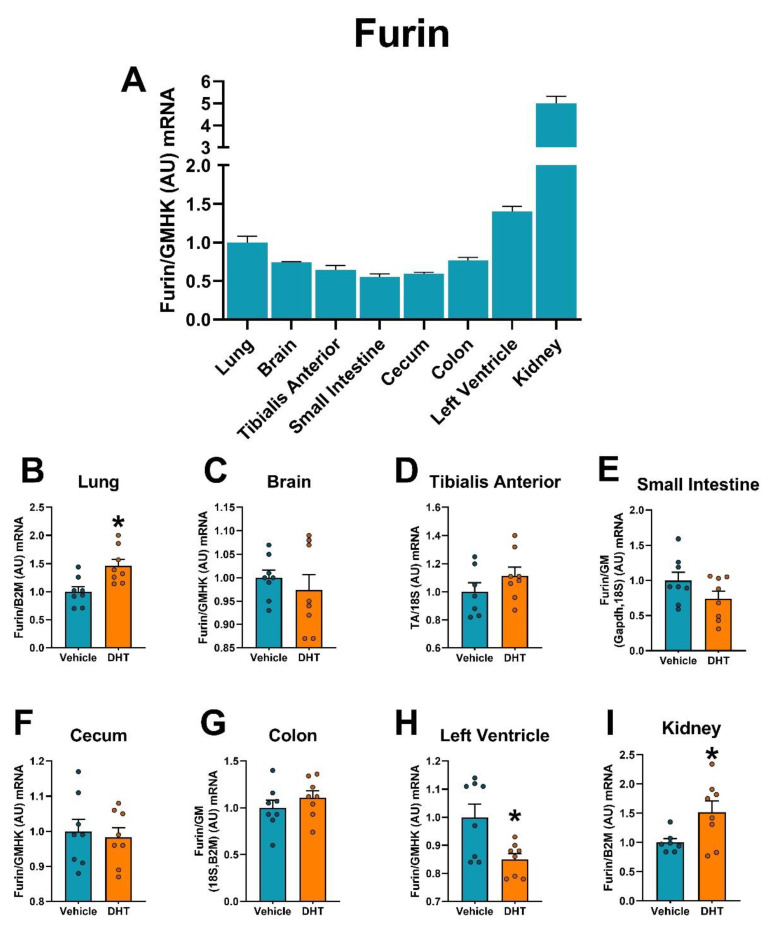
Furin mRNA expression regulation by DHT in different tissues. Furin mRNA expression was quantified by RT-qPCR. (**A**) Furin mRNA expression in control mice normalized to the geometric mean of all reference genes and expressed relative to lung tissue. (**B**–**I**) Furin mRNA expression in the lung (**B**), brain (**C**), tibialis anterior (**D**), small intestine (**E**), cecum (**F**), colon (**G**), left ventricle (**H**), and kidney (**I**) of control and DHT-treated mice normalized to the least variable reference gene or set of them and expressed relative to control mice. Data analyzed by *t*-test. *n* = 7–8/group. *: *p* < 0.05.

Similar to the protease Cathepsin L, relative mRNA expression of Furin was highest in the kidney, showing ~5-fold higher expression than the lung (Figure 9A). Furin was upregulated in DHT-treated animals in the lung (1.46-fold, Figure 9B) and the kidney (1.69-fold, Figure 9I), and downregulated in DHT-treated animals in the left ventricle (0.85-fold, Figure 9H).

**Figure 10:**
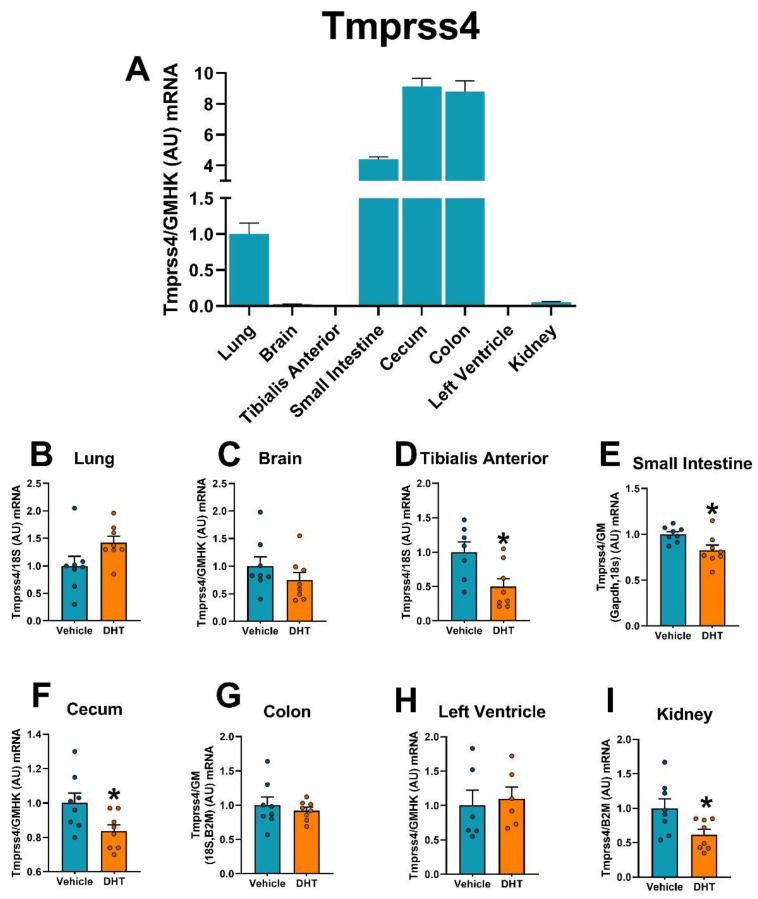
Tmprss4 mRNA expression regulation by DHT in different tissues. Tmprss4 mRNA expression was quantified by RT-qPCR. (**A**) Tmprss4 mRNA expression in control mice normalized to the geometric mean of all reference genes and expressed relative to lung tissue. (**B**–**I**) Tmprss4 mRNA expression in the lung (**B**), brain (**C**), tibialis anterior (**D**), small intestine (**E**), cecum (**F**), colon (**G**), left ventricle (**H**), and kidney (**I**) of control and DHT-treated mice normalized to the least variable reference gene or set of them and expressed relative to control mice. Data analyzed by *t*-test. *n* = 7–8/group. *: *p* < 0.05.

Similar to Tmprss2 mRNA, the gastrointestinal tract showed the highest expression for the protease Tmprss4, relative to the lung (Figure 10A). Tmprss4 mRNA was downregulated in DHT-treated animals in the tibialis anterior muscle (0.50-fold, Figure 10D), the small intestine (0.75-fold, Figure 10E), the cecum (0.84-fold, Figure 10F) and the kidney (0.62-fold, Figure 10I).

**Figure 11:**
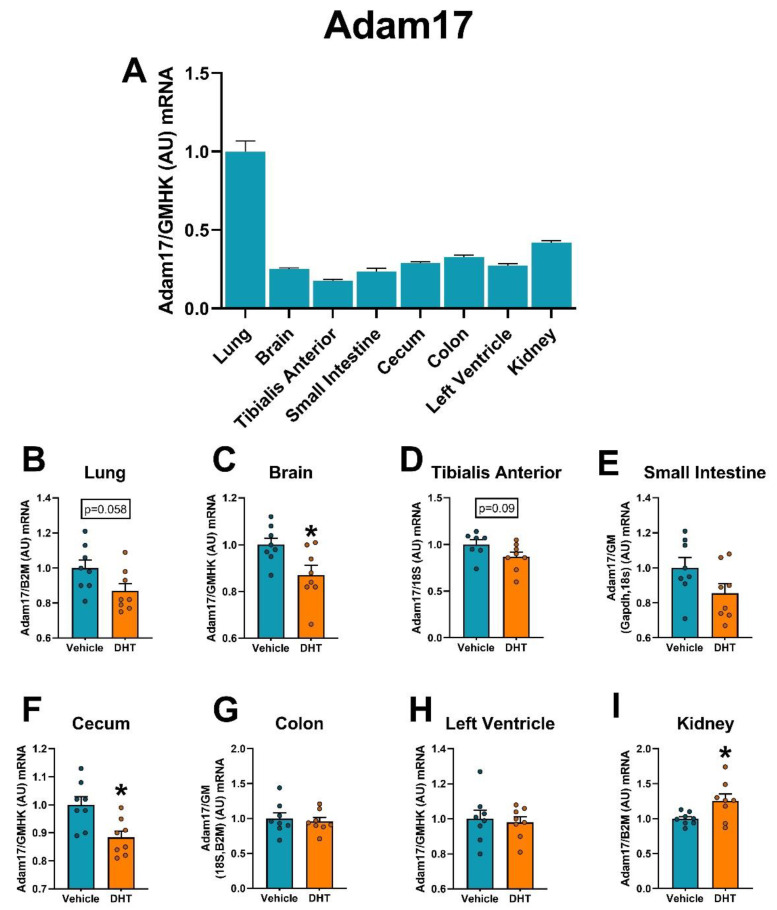
Adam17 mRNA expression regulation by DHT in different tissues. Adam17 mRNA expression was quantified by RT-qPCR. (**A**) Adam17 mRNA expression in control mice normalized to the geometric mean of all reference genes and expressed relative to lung tissue. (**B**–**I**) Adam17 mRNA expression in the lung (**B**), brain (**C**), tibialis anterior (**D**), small intestine (**E**), cecum (**F**), colon (**G**), left ventricle (**H**), and kidney (**I**) of control and DHT-treated mice normalized to the least variable reference gene or set of them, and expressed relative to control mice. Data analyzed by *t*-test. *n* = 7–8/group. *: *p* < 0.05.

Lung Adam17 mRNA expression showed the highest expression of all of the tissues (Figure 11A). Comparing individual tissues, expression of the sheddase Adam17 mRNA was downregulated in DHT-treated animals compared to control in the brain (0.87-fold, Figure 11C) and the cecum (0.88-fold), Interestingly, the kidney showed increased Adam17 expression in DHT-treated animals compared to controls (1.25-fold, Figure 11I).

## 3. Discussion

Our study aimed to evaluate the regulation of SARS-CoV-2 viral entry proteins by androgens in a mouse model of PCOS. Given that both COVID-19 and PCOS affect multiple overlapping systems in the body, we attempt to highlight key potential vulnerabilities in this population. 

In addition to its role as a receptor for SARS-CoV-2, ACE2 is also an enzyme that generates angiotensin (1–7) (Ang (1–7)). ACE2 is a carboxypeptidase that is an integral membrane-bound protein, which inactivates the vasoconstrictor peptide angiotensin II (Ang II) by its cleavage to Ang (1–7). The ACE2/Ang (1–7)/Mas receptor axis, the non-classical arm of the Renin-Angiotensin System (RAS), is cardioprotective since it opposes the ACE/Ang II/AT1R axis, the classical arm of the RAS, and leads to reduced inflammation and fibrosis with increased vasodilation in the heart [32]. Several studies indicate that perturbation in the RAS can increase blood pressure in both women with PCOS [33] and in hyperandrogenemic female animal models [34]. In our study, both mRNA and protein levels of ACE2 were increased in the kidney and the left ventricle, as well as increased ACE2 protein in the small intestine in hyperandrogenemic females. This highlights the potential importance of ACE2 in these tissues, which may be upregulated to counteract the RAS overactivity in hyperandrogenism. ACE2 upregulation may also indicate that these tissues may be more susceptible to SARS-CoV-2, due to the potential for increased viral entry and subsequent replication. Transient upregulation of ACE2 has been shown initially with low viral titer infections [35]. However, following viral entry of SARS-CoV, and likely SARS-CoV-2, ACE2 is downregulated by internalization, whereby it is no longer functional [36,37]. In this way, any increased protection formerly provided by ACE2 to compensate against RAS stimulation in hyperandrogenism would not be beneficial after SARS-CoV-2 infection. 

In the lung, ACE2 deficient mice have more severe acute lung injury promoted by an increase in Ang II production acting through the AT1R [38]. Treatment with AT1R blockers along with recombinant ACE2 were able to attenuate the lung injury in SARS-CoV, showing the importance of ACE2 in acute lung injury [36]. We found increases in Ace2 mRNA in the lung with DHT but did not find increases at the protein level, thus women with PCOS may not be at a greater risk for lung-specific damage from COVID-19. However, given the systemic RAS imbalance observed in women with PCOS without enhanced ACE2 protection in the lung, it is possible that maintenance of pulmonary homeostasis and appropriate oxygenation following SARS-CoV-2 viral entry may not be adequate to combat infection in these women. In our study, both the lung and the kidney had increased mRNA expression of the protease Furin, which is instrumental in the activation of the SARS-CoV-2 S-protein to infect human lung airway cells [39]. Interestingly, there is evidence of a connection between elevated circulating furin and metabolic dysfunction, potentially in order to compensate for increased synthesis of active insulin receptors, which are cleaved by furin [40]. Because furin is shed from most cells and also has similar activity in its membrane-bound form, it may be an interesting target in PCOS and COVID-19. A recent report showed that female mice treated with testosterone for 5 days have no change in lung Ace2 or Tmprss2 mRNA or protein expression [10]. However, in our study, we observed an increase in lung Ace2 mRNA with DHT treatment. There are several possible explanations for such discrepancies. DHT is a non-aromatizable androgen while testosterone can be converted to estrogens by the action of the aromatase, particularly in female animals. Since androgens and estrogens have opposite effects in multiple physiological processes, the use of the pure androgen DHT allows for parsing the effect of androgens alone on lung ACE2 expression in our model. Another plausible explanation is that we performed a chronic 3-month long DHT treatment, compared with an acute effect examined after only 5 days of testosterone. The effects observed in our model better mimic the physiological effects observed in chronic diseases such as PCOS.

In our study, hyperandrogenemic female mice showed decreased Adam17 mRNA expression in the brain and increased expression in the kidney. Adam17 is a disintegrin and metalloprotease, also called a TNF-α converting enzyme (TACE), which can cleave the ectodomain of ACE2 from its membrane-bound state to its soluble form. The brain has reduced expression of Adam17 and ACE2 mRNA with DHT treatment, although ACE2 downregulation was not significant at the protein level. ACE2 downregulation could be beneficial by decreasing susceptibility to SARS-CoV-2 infection but, on the other hand, ACE2 downregulation could translate into reducing protective effect by the ACE2/Ang (1–7)/Mas pathway. Some of the neurological effects observed in COVID-19 include headache, dizziness, hyposmia, seizures, and stroke [41]. Following the first days after ischemic stroke, components of the ACE2/Ang (1–7)/Mas increase in human serum [42] and in rat ischemic cortex lesions [43]. Ace2 gene expression in the brain is important for the regulation of blood pressure and decreases with age, specifically in males but not females [35]. PCOS is associated with an increased risk of psychiatric disorders including binge-eating disorder depression, anxiety, and sleep disorders [44,45]. In a large cohort of COVID-19 survivors, the incidence of those same psychiatric disorders in patients was increased by 18% in the first three months following recovery [46]. Additionally, in a Chinese cohort, almost 36% of patients had neurological symptoms, and COVID-19 disease severity was associated with increased neurological dysfunction [47]. Although women with PCOS may not have increased susceptibility to SARS-CoV-2 infection in the brain based on viral entry protein expression, it is possible to speculate that decreased Adam17 expression in the brain could impact the neurological disorders observed in PCOS.

The tibialis anterior muscle, composed of fast-twitch muscle fibers, relies on anaerobic respiration for short-term endurance. The skeletal muscle is an insulin-sensitive organ that stores and uses glucose as a fuel source. Studies in animal models of PCOS [48] and women with PCOS [49] indicate that hyperandrogenemia increases insulin resistance in the skeletal muscle and increased lean mass. Expectantly, animals in our study showed an increase in lean mass with DHT treatment. Given the prevalence of insulin resistance in hyperandrogenemic PCOS is estimated in approximately 40–70% of women [50] and that the nervous and musculoskeletal systems are targets in COVID-19 infection, the skeletal muscle’s androgen-mediated impairment of glucose homeostasis may be a relevant cofactor in metabolic risk for COVID-19. Although we did not find significant TMPRSS2 protein level changes, we found significant mRNA downregulation in the proteases relevant in cleavage of SARS-CoV-2 virus in the muscle, namely Cathepsin L and Tmprss4. Transmembrane serine proteases (TTSPs) and furin act at the cell surface to activate the coronavirus spike proteins whereas Cathepsin L activates the S-protein for fusion by endocytosis [1]. This priming is essential to release the virus into the cytoplasm. Recently, Cathepsin L inhibitors have been reported to decrease entry of SARS-CoV-2 in 293 cells expressing human ACE2 [51]. Impaired Cathepsin L expression in the skeletal muscle is associated with Type 2 Diabetes and insulin resistance both in animals and humans [52]. Therefore, the observed decrease in Cathepsin L in skeletal muscle, a tissue already at high risk for androgen-mediated insulin resistance, is more likely a consequence of the insulin resistance that characterizes our PCOS model. A myriad of inflammatory cytokines including TNF-α, IL-6, and C-reactive protein (CRP), are increased in skeletal muscle under conditions of insulin resistance [53]. Although downregulation of Cathepsin L and Tmprss4 may decrease viral entry in the skeletal muscle in women with PCOS, the suspected increase in local inflammation in the muscular tissue in these women raise a possibility of greater vulnerability to tissue injury following systemic cytokine storm in SARS-CoV-2 infection.

Interestingly, our results show that DHT-treated female mice have pronounced changes to the protein expression of ACE2 and TMPRSS2 in the small intestine, in addition to having the highest Ace2 mRNA expression relative to all other tissues analyzed. Besides its role in viral entry, ACE2 expression in the luminal epithelial cells of the intestine is required for adequate nutrient uptake [54]. Additionally, the TMPRSS4 protease, besides TMPRSS2, promote SARS-CoV-2 infection in human enterocytes [55]. Consequently, the small intestine may be a compelling target of COVID-19 related issues in women with PCOS. Androgen metabolism and signaling are important throughout the gastrointestinal tract. Microbiota in the distal region of the small intestine are responsible for the deconjugation of DHT and testosterone [56]. Specifically, we used the jejunum in this study, which is where digestion primarily takes place. Interestingly, female mice who were transplanted with the cecal contents of the male gut had metabolic changes and a subsequent increase in their serum testosterone [57]. This further supports the importance of androgens in digestion, in addition to sex-specific differences in other systemic responses [58]. 

Gut integrity of the intestinal barrier acts as a first line of defense against the entry of pathogens and harmful substances into the circulation. The gut microbiome exhibits functional activities in its coordination with the host to influence immunity and inflammatory response [59]. Recently, the gut microbiota was shown to influence signaling to lung epithelia following viral influenza in order to enhance resistance to infection [60]. A number of recent studies indicate that women with PCOS have a different gut microbiome composition than healthy women and hyperandrogenism is a primary driver of dysbiosis in women with PCOS [61,62]. Moreover, patients with COVID-19 display an altered gut microbiome profile, having reduced commensal bacteria and increased pathogens following viral infection [63]. Women with a hyperandrogenemic phenotype of PCOS, who already may have dysbiosis, may be at even greater risk for longer-term consequences following COVID-19 related to metabolic homeostasis given the potential impact on the small intestine. Additionally, in the colon, mRNA expression of Tmprss2 was higher in our DHT female mice, which could suggest potential increased viral entry of SARS-CoV-2 in this tissue. However, on the other hand, the colon had reduced Ace2 mRNA expression with DHT treatment. Functional analyses indicate that Ace2 expression in the colon is associated with fatty acid metabolism [35], indicating that this major metabolic pathway may be impaired in women with PCOS. Although acute respiratory system dysfunction is the primary symptom of COVID-19, the presence of digestive system disruptions in COVID-19 is increasingly recognized [64]. Moreover, a recent meta-analysis of patients with COVID-19 presenting with gastrointestinal symptoms on admission showed a higher risk of respiratory, cardiac, and renal complications, including increased mortality [65]. Women with PCOS have an increased risk of other gastrointestinal issues including irritable bowel syndrome [66,67], which may itself portend a higher risk of infection [64]. The potential for exacerbation of gastrointestinal tract related issues following COVID-19 in women with PCOS may place these women at high risk for severe complications.

Comorbidities that place individuals at higher risk for severe COVID-19 coincide with those of PCOS including diabetes and obesity [68]. In our study, we observed metabolic disturbances as increases in body weight, fat mass, fasting glucose, and perturbed glucose homeostasis. Recent metabolomic studies suggest that profiling of carbohydrates, lipids, amino acids, nucleotides, and steroid metabolites can help to gain further insight into the metabolic derangements present in women with PCOS [69,70]. Women with PCOS have a ~3-fold greater risk of developing type II diabetes compared with healthy women [71]. Other cardiometabolic risk factors in PCOS include alterations to blood lipid profile and increased risk for non-fatal cerebrovascular disease, which interferes with blood flow to the brain [71]. COVID-19 not only leads to higher mortality in individuals with existing cardiovascular disorders and hypertension [35], but the virus can also be the cause of these ailments in those patients [72]. In addition to physiological changes, at the molecular level, we observed increases in Ace2 at both the mRNA and protein level in the left ventricle of the heart. As mentioned above, the increase in ACE2 not only represents a potential increase in viral entry but also exhibits the underlying disturbance to the RAS system and an attempt to counterbalance this issue by upregulation. A myriad of cardiac dysregulations has been reported in COVID-19 patients ranging from limited necrosis of heart cells, to myocarditis and even to cardiogenic shock [73]. However, the almost negligible TMPRSS2 expression in cardiac tissue has led to doubts that the heart is a direct target of SAR-CoV-2 infection. Recent studies have shown that other S-protein proteases, such as cathepsin L and furin, are highly expressed in multiple types of cardiac cells [31]. Both furin and cathepsin L prime SAR-CoV-2 for cell infection [51,74]. The relatively high expression of those proteases in cardiac tissue, as observed in our model of PCOS, would provide an alternative route for SAR-CoV-2 for cell infection bypassing Tmprss2 and help explain cardiac pathology in COVID-19.

Among the cardiometabolic perturbations in women with PCOS, increased blood pressure in these women is of primary relevance to disturbances in renal outcomes. Not only is hypertension the leading risk factor for the development of cardiovascular disease, but it is one of the leading causes and consequences of kidney failure [75]. Women with PCOS may be at increased risk of development of renal injuries as reported by marked alterations to kidney function in animal models [34,76]. Importantly, acute kidney injury is observed in COVID-19 [77], in which approximately 20–40% of intensive care COVID-19 patients suffer severe kidney injury [78]. Moreover, we recently postulated that androgens, via alterations in the intrarenal renin-angiotensin system, impair renal hemodynamics, predisposing patients to acute kidney injury during COVID-19 infection, which could explain the higher mortality observed in men with COVID-19 [79]. The kidney was the most dysregulated tissue in hyperandrogenemic females in our study in terms of expression alterations. Renal ACE2 mRNA and protein were upregulated in DHT-treated female mice suggesting an increased risk for COVID-19-related renal complications in women with PCOS. Among the proteases, TMPRSS2, Cathepsin L, and Furin mRNA were upregulated in the kidney of DHT-treated female mice suggesting an increased susceptibility to SARS-CoV-2 renal infection in women with PCOS. The Epithelial sodium channels (ENaC) regulate sodium absorption; the TMPRSS4 protease can activate these channels by cleavage of different ENaC subunits which may be relevant for the maintenance of normal kidney function [80]. ENaC channel activity is stimulated by insulin in the kidney [81]; this may be an important link between kidney injury and the hyperinsulinemia observed in PCOS, enhanced by dysregulation in TMPRSS4. Together with the increased expression of the SAR-CoV-2 receptor ACE2, our findings show that the kidney present upregulation of multiple SARS-CoV-2 viral entry proteins suggesting a need for closer follow-up of renal injury and function in women with PCOS.

It has been suggested that ACE2 is actively shed in the urine by Adam17 proteolytic activity, although it is undetermined if the proximal tubule cells are the primary source. Increases in urinary ACE2 have been found in type II diabetic patients [82] and patients with diabetic nephropathy [83]; it correlated with both metabolic and renal parameters. In our study, we did not find differences in circulating serum ACE2, though decreased serum Ace2 has been shown recently in PCOS patients along with increased serum renin [33]. Our findings of increased urinary ACE2, without significant changes in circulating ACE2 in the PCOS animal model, are similar to the findings from a study of chronic kidney disease patients [84]. Because these patients had high albuminuria caused by kidney injury, it may be interpreted that there is leakage of the ACE2 across the glomerular barrier. Although we did not assess albuminuria, our DHT-treated female mice did show an elevation in fasting blood glucose compared with controls, which has also been associated with urinary ACE2 [85]. Additionally, as aforementioned, androgen-mediated increases to the ACE2 sheddase Adam17 mRNA were observed in the kidney in our study, which may account for the higher urinary levels of ACE2 in these animals by mediating its shedding. Interestingly, increased ADAM17 is associated with type II diabetes [82]. Up to now, there have been no previous reports of regulation of urinary ACE2 in women with PCOS nor in DHT-treated animal models of PCOS.

**Figure 12:**
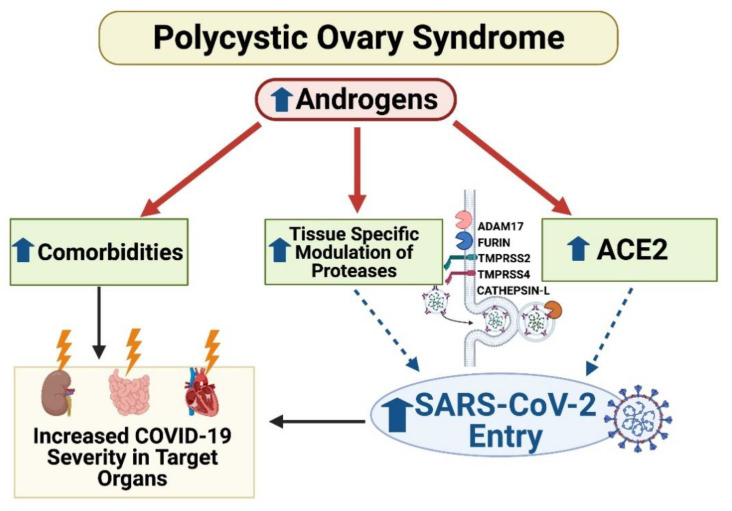
Postulated mechanism of increased SARS-CoV-2 infection and worsened clinical outcomes in PCOS. In PCOS, elevated androgens upregulate the SARS-CoV-2 receptor ACE2 and modify host proteases to increase SARS-CoV-2 viral entry into tissues. Target organs at greatest potential risk primarily include the kidney, the small intestine, and the heart. In conjunction with enhanced viral entry, comorbidities that place individuals at high risk for COVID-19 disease severity coincide with those associated with PCOS, adding to their predisposition for worsened COVID-19 outcomes. Up arrow signifies increase(s); lightning bolt represents injury.

In summary, the results of our study identify women with PCOS as a population at potentially higher risk of disease severity from COVID-19. The differences observed in androgen-treated mice highlight that several mechanisms involved in SARS-CoV-2 entry may be altered in women with PCOS and translate to the reduced protection of specific target organs known to be susceptible to COVID-19 injury (Figure 12). Health care providers may be advised to focus on renal, cardiac, and gastrointestinal aspects in PCOS patients, as these may be systems with the highest potential for COVID-19 severity in this patient population.

## 4. Materials and Methods

### 4.1. Animals 

Four-week-old C57BL/6N female mice (Charles River Laboratories, Wilmington, MA, USA) were implanted subcutaneously with a SILASTIC tube (1.5 cm; id, 1.47 mm; od 1.95 mm, #508-006 Dow Corning, Midland, MI, USA) containing 8.0 mg dihydrotestosterone (DHT, Steraloids Inc., Newport, RI, USA) or empty tubes to generate a well-established mouse model hyperandrogenemic PCOS [86]. The peripubertal DHT exposure animal model of PCOS elicits an adult phenotype that exhibits a breadth of endocrine, reproductive, and metabolic traits that closely resemble the human pathology [87]. Animals were treated with DHT or control for 90 days. Animals of the same treatment were housed together in a 12:12 h light-dark cycle under temperature-controlled conditions. Animals were fed ad libitum throughout the experiment on a regular chow diet (Teklad global 2018, 18% protein rodent diet, Envigo, Indianapolis, IN, USA). For the oral glucose tolerance test (OGTT), 5 h fasted animals were administered with an oral glucose load (2 g glucose per kg lean mass wt) by gavage, and blood glucose levels were measured at 0, 15, 30, 60, 90, and 120 min with a standard glucose meter (ContourNext, Bayer, Leverkusen, Germany) from the tail vein. After a 3-day recovery period, a fasting blood sample was obtained by facial vein lancet in 5 h fasted animals. Fat and lean mass were assessed using an EchoMRI 4in1 body composition analyzer. Animals were euthanized by left ventricle exsanguination and organ removal under isoflurane after 90 days of treatment and perfused with 10 mL of sterile saline. All experimental protocols were performed in accordance with the National Institutes of Health’s Guide for the Care and Use of Laboratory Animal 8th edition (2011), and reviewed and approved by the Institutional Animal Care and Use Committee of the University of Mississippi Medical Center. Tissues were weighed and flash-frozen in liquid nitrogen and stored at −80 °C. Tissues collected include the lung, kidney, left ventricle, left hemisphere of the brain, the tibialis anterior muscle, the jejunum, the cecum, and the proximal colon.

### 4.2. RNA and RT-qPCR

Extraction of total RNA was performed using TRI-Reagent (Molecular Research Center Inc., Cincinnati, OH, USA) following recommended protocol by the manufacturer. RNA samples were resuspended in nuclease-free water and underwent routine DNAse-free treatment following the manufacturer’s protocol (Ambion TURBO DNA-free, ThermoFisher Scientific, Waltham, MA, USA). RNA concentrations were quantified using UV absorbance on a microvolume spectrophotometer (NanoDrop Technologies, ThermoFisher Scientific, Waltham, MA, USA). Reverse transcription was carried out using 1.75 µg total RNA with SuperScript IV Reverse Transcriptase (ThermoFisher Scientific, Waltham, MA, USA) for cDNA synthesis. Gene expression was performed using real-time quantitative polymerase chain reaction (RT-qPCR) with TaqMan technology with Luna Universal Probe qPCR MasterMix (New England BioLabs, Ipswich, MA, USA) on a QuantStudio3 system. The following primer/probe pairs (Applied Biosystems, Waltham, MA, USA) were used: Ace2 (Mm01159006_m1), Adam17 (Mm00456428_m1), Ctsl (Mm00515597_m1), Furin (Mm00440646_m1), Tmprss2 (Mm00443687_m1), Tmprss4 (Mm00520486_m1), Actb (Mm02619580_g1), B2m (Mm00437762_m1), Gapdh (Mm99999915_g1), 18S rRNA (Hs99999901_s1). Relative expression to the geometric mean of reference genes (ActB, B2m, Gapdh, 18S rRNA) CT values was used and data is shown in arbitrary units relative to the vehicle group. 

### 4.3. Western Blot

Tissue samples (10–50 mg) were homogenized in radioimmunoprecipitation assay (RIPA) buffer containing Halt protease and phosphatase inhibitor cocktail (Thermo Scientific, Waltham, MA, USA). Total protein was quantified with Pierce bicinchoninic acid protein assay kit (Thermo Scientific, Waltham, MA, USA). Protein were separated by sodium dodecyl sulfate-polyacrylamide gel electrophoresis using TGX Stain-Free Gels (Bio-Rad, Hercules, CA, USA) and transferred to polyvinylidene fluoride membranes. Blocking with 5% nonfat dry milk in Tris-buffered saline containing 0.1% Tween 20 was performed on membranes for 1 h at room temperature and then incubated overnight at 4 °C with the following primary antibodies: anti-Angiotensin I converting enzyme 2 (Abcam ab108252, Cambridge, MA, USA); anti-Androgen receptor (Abcam ab133273, Cambridge, MA, USA); anti-Tmprss2 (Abcam ab92323, Cambridge, MA, USA); anti-Cathepsin L (Invitrogen MA5-23891, Waltham, MA, USA). Membranes were probed with horseradish peroxidase-conjugated goat anti-rabbit or anti-rat IgG secondary antibodies (Cat No. 111-035-003 and 112-035-003, Jackson ImmunoResearch Laboratories, West Grove, PA) for 1 h at room temperature. Chemiluminescence detection was performed on a ChemiDoc MP imaging system (Bio-Rad, Hercules, CA, USA) using SuperSignal West Pico PLUS kit (ThermoFisher Scientific, Waltham, MA, USA) as substrate. For normalization, membranes were stripped with Restore Western Blot Stripping Buffer (ThermoFisher Scientific, Waltham, MA, USA), incubated overnight at 4 °C with anti-GAPDH antibody (1:300,000; Cat No. 5174, Cell Signaling Technology, Danvers, MA, USA), and processed as described above. Quantification and background subtraction analyses were carried out using Fiji ImageJ software version 1.53c [88].

### 4.4. Serum and Urine ACE2

Serum was obtained from blood after one-hour incubation at room temperature and centrifugation at 1300× *g* for 10 min at 4 °C. Urine was collected from animals placed in 24-h metabolic cages after 12 weeks of treatment. ACE2 ELISA was performed diluting the urine or serum sample 1:2 in sample diluent and following a standard protocol (Abcam ab213843, Cambridge, MA, USA). Urine samples were corrected by urinary creatinine, which was determined using an automated chemical analyzer (Vet Axcel, Alfa Wassermann Diagnostic Technologies, West Caldwell, NJ, USA).

### 4.5. Statistics

All data are presented as mean ± SEM with statistical differences considered to be *p* < 0.05. Student’s t-test was performed for comparison of two groups, with Welch’s correction for samples with unequal variances. Assumptions on normality and equal variance were checked using Shapiro–Wilk and F tests. All analyses were performed in Prism 8 software version 8.4.3 (GraphPad Software Inc., La Jolla, CA, USA).

## Data Availability

Data is contained within the article or Supplementary Material.

## Supplementary Materials

The following are available online at https://www.mdpi.com/article/10.3390/ijms22094472/s1, Figure S1: Cathepsin L protein expression regulation by DHT in the left ventricle.
