# Supplementary material for: SARS-CoV-2 Viral Entry Proteins in Hyperandrogenemic Female Mice: Implications for Women with PCOS and COVID-19
Source: Int J Mol Sci. 2021 Apr 25;22(9):4472. doi: 10.3390/ijms22094472 (PMC8123333; doi:10.3390/ijms22094472)
Supplement: Supplementary file 1 [file ijms-22-04472-s001.zip › ijms-1151290-supplementary.pdf]

Supplemental Figure 1

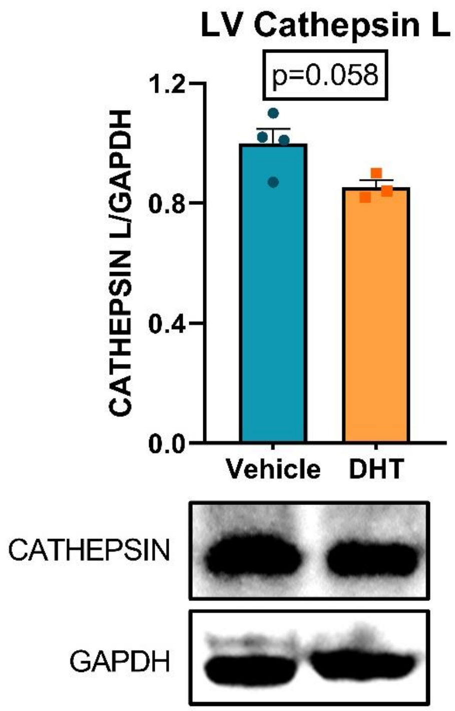

Supplemental Figure 1. Cathepsin L protein expression regulation by DHT in the left ventricle. Protein expression was normalized to GAPDH, and expressed relative to control mice. Data analyzed by t-test. n=3-4/group. *: $p < 0.05$.
